# Bezold’s abscess, an uncommon complication of otitis media and cholesteatoma: a case report

**DOI:** 10.1186/s13256-024-04631-y

**Published:** 2024-07-12

**Authors:** Alexandra J. Toloczko, Thomas S. Davis, Mayada H. Issa

**Affiliations:** 1 Virginia Tech Carilion School of Medicine, Roanoke, VA USA; 2 Department of Internal Medicine, Carilion Clinic, Roanoke, VA USA

**Keywords:** Bezold abscess, Otitis media, Mastoiditis, Cholesteatoma

## Abstract

**Background:**

Mastoiditis frequently occurs in children as they are more susceptible to middle ear infections, but infrequently occurs in adults. A rare complication that results from mastoiditis and an obstructing cholesteatoma is a Bezold’s abscess, of which there are less than 100 reported cases in literature to date.

**Case presentation:**

Here, we present a case of a 72-year-old Caucasian man who has had no history of prior ear infections and was found to have a cholesteatoma and advanced acute coalescent mastoiditis complicated by a Bezold’s abscess.

**Conclusions:**

Bezold’s abscess is a rare entity infrequently encountered in the modern era, likely owing to more prompt treatment of otitis media. Cholesteatoma poses a great risk for both the development of otitis media and further progression to mastoiditis and its associated complications, such as Bezold’s abscess. Knowledge of said abscess is crucial; without prompt recognition, further spread of infection can occur with vascular or mediastinal involvement.

## Background

Mastoiditis is inflammation of a portion of the temporal bone and commonly results from the progression of acute otitis media [1]. Incidence of mastoiditis from acute otitis media is reported as 0.004% in the US [2]. There are three different categories of mastoiditis, which are based on the extent of invasion. Incipient mastoiditis is an infection of the mastoid air cells alone with no continuation into the middle ear cavity [1]. Subacute mastoiditis is persistent middle ear infection with inadequate antimicrobial therapy, which results in erosion of the boney septations between mastoid cells [1]. Acute coalescent mastoiditis is characterized by inflammation of the epithelial lining with erosion through the bony septations of the mastoid air cells, which can lead to the coalescence of small air cells into larger ones full of pus, and thus abscess formation [1]. One rare abscess that results from coalescent mastoiditis is a Bezold’s abscess. These are rare deep neck abscesses where the infection erodes through the lateral mastoid complex medial to the attachment of the sternocleidomastoid muscle, of which there are only 100 cases reported to date since the 1960s [1, 3]. Bezold’s abscesses can be found at any age, and are more commonly found in males.

Cholesteatoma is a benign collection of keratinized squamous epithelium that affects various spaces, such as the middle ear, mastoid, or petrous bone, and is known for its tendency to recur [4]. If not properly managed it can result in both intracranial and extracranial complications. Intracranial complications include meningitis, brain abscess, sigmoid sinus involvement, extradural or subdural abscess, and hydrocephalus [5]. The extracranial complications include a mastoid abscess, subperiosteal abscess, petrositis, labyrinthine fistula, and Bezold abscess [5]. The association between cholesteatoma and Bezold abscess is infrequently reported owing to the widespread use of antibiotics preventing the aforementioned complications [5].

The oral microbiota is made up of approximately 1000 species of bacteria, including the phyla Actinobacteria, Bacteroidetes, Chlamydia, Euryarchaeota, Fusobacteria, Firmicutes, Proteobacteria, Spirochaetes, and Tenericutes [6]. The concentrations of these bacteria vary widely based on the location in the oral cavity, stage of dentition, and comorbid illnesses [6]. The most common pathogens in acute mastoiditis are *Streptococcus pneumoniae*, *S*. *pyogenes*, *Staphylococcus aureus*, and *Haemophilus influenzae* [1]. In patients with chronic otitis media and with a cholesteatoma, the most frequent anaerobes were *Peptostreptococcus*, *Fusobacterium*, *Prevotella*, and *Porphyromonas* [7]. Although mastoiditis can occur at any age, children are more susceptible to middle ear infections, thus they are at a greater risk of developing acute mastoiditis compared with adults. However, the average age of patients presenting to the emergency room with complications of otitis media is 37 years of age [8]. Other risk factors include an immunocompromised state, recurrent acute otitis media, or incomplete pneumatization of the mastoid process [1].

If Bezold’s abscesses are present or suspected it is necessary to start broad spectrum antibiotic therapy with good cerebrospinal fluid penetration and obtain appropriate imaging to evaluate the location and size of the abscess collection [9]. Additionally, early surgery is necessary to establish drainage of the middle ear and mastoid cells. If a deep neck fluid collection exists concurrently with mastoiditis, a complete mastoidectomy should be performed. Finally, its crucial to sample the purulent material to replace initial broad-spectrum antibiotics with ones to which the pathogen has known susceptibility [10].

This report is significant as most of the literature focuses on pediatric cases, and this case report details a case of a man in his 70s who presented to the emergency room after weeks of worsening right ear pain, which ultimately proved to be an advanced acute coalescent mastoiditis complicated by a Bezold’s abscess.

## Case presentation

The patient was a 72-year-old Caucasian male with a past medical history significant for hypertension, hyperlipidemia, coronary artery disease with two prior stents, more than 10 pack years of smoking history, and chronic obstructive pulmonary disease (COPD) who presented to the emergency department (ED) with 3 weeks of worsening right ear pain. The patient initially presented to his primary care physician for the ear pain and on physical exam the ear canal was tender with swelling and a limited amount of yellow-colored discharge. The tympanic membrane was not injected, scarred, perforated, or erythematous. The patient had chronic hearing loss from presbycusis bilaterally, so hearing aids were in place but had no signs of sensorineural hearing loss. Ultimately, the patient was diagnosed with acute otitis externa of his right ear and was prescribed otofloxacin ear drops. However, 5 days into his 10-day course the patient began experiencing non-bloody drainage from the right ear and swelling behind/in front of the ear. At this time the patient decided to come to the ED. Other associated symptoms included weakness, fatigue, and dyspnea on exertion that had progressed during this time. The patient did not have any changes in his baseline cough and denied fever, chills, chest pain, headache, visual changes, nasal congestion, sore throat, nausea, and vomiting. The patient also denied similar symptoms on the left side. The rest of the review of systems was unremarkable.

The patient’s vital signs upon arrival to the department indicated that he was afebrile at 98.1° Fahrenheit, normotensive with a blood pressure of 98/65 mmHg, sinus tachycardic with a heart rate of 140–150 beats per minute, respiratory rate of 24 breaths per minute, and saturating at an SPO2 of 94% on 2L nasal cannula.

Upon physical exam, the patient was an acutely ill-appearing male in mild distress owing to right ear pain. There was crusted drainage from the right ear, edema, and erythema overlying the mastoid bone, and a round tender mass with fluctuance inferior to the lateral mandible overlaying the sternocleidomastoid. A bedside ear, nose, and throat (ENT) exam showed similar tympanic membrane findings to those previously mentioned. Furthermore, the patient was tachycardic, with no murmur, and had bilateral wheezing in all fields. The extremities showed trace edema bilaterally in the lower extremities. Cranial nerves 2 through 12 were intact, and the patient showed no signs of meningismus.

His initial workup revealed leukocytosis with 31,000 cells/mcL and a high neutrophil band percentage of 11%. The complete metabolic panel (CMP) showed hyponatremia with 127 mEq/L and hyperglycemia with 166 mg/dl. It should be noted that the patient had a normal A1c (5.4) 1 month prior to this admission, so we suspected the hyperglycemia to be stress-induced. The remainder of the lab values were normal or nonpertinent to diagnosis. Methicillin-resistant *Staphylococcus aureus *(MRSA) nares and viral polymerase chain reaction (PCR) were also collected in the ED and were negative, and blood cultures showed no immediate results.

**Fig. 1:**
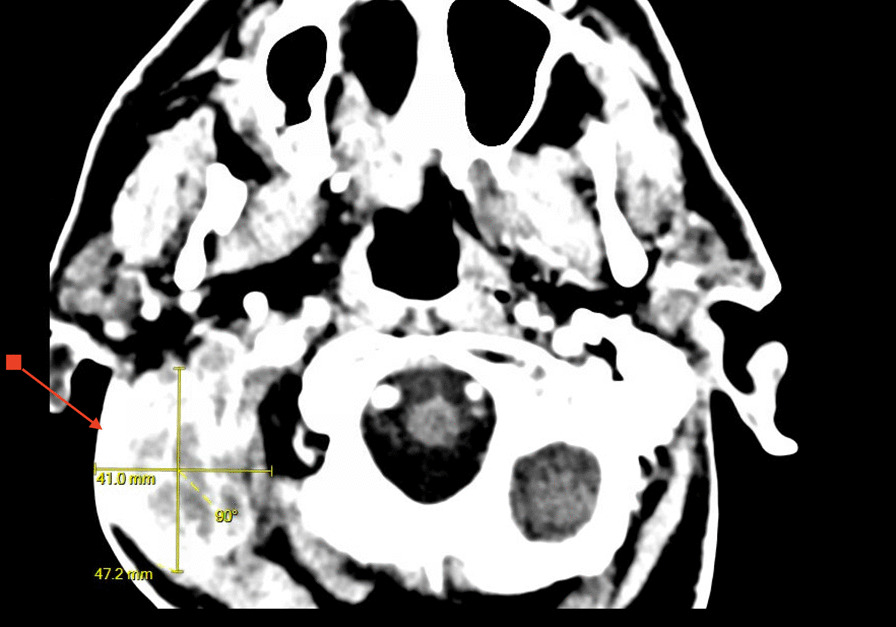
Heterogeneous 4.1 × 4.7 cm mass (red arrow) adjacent to right mastoid air cells

**Fig. 2:**
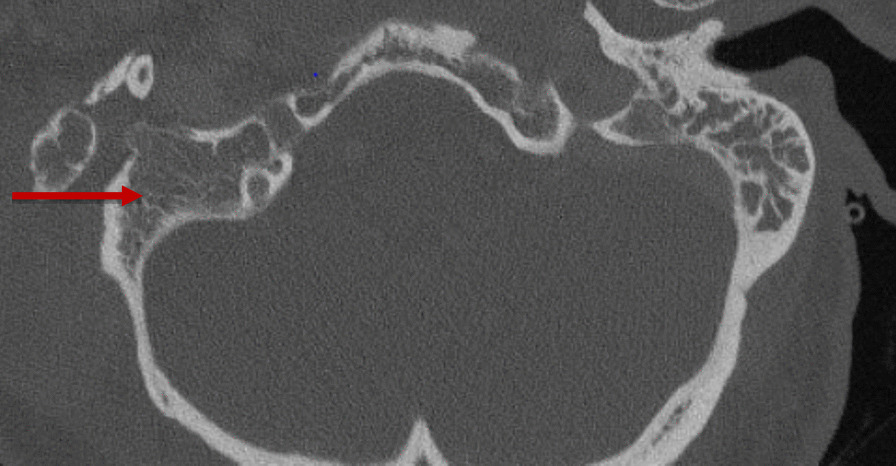
Osseous destruction (red arrow) of the right mastoid bone with opacification of the right mastoid air cells

A noncontrast computed tomography (CT) scan of the temporal bones and a chest X-ray were ordered in the ED. The CT showed coalescent right acute mastoiditis with a subperiosteal abscess measuring 4.1 × 4.7 cm (Bezold abscess) without evidence of extension into the posterior fossa or dural venous sinus thrombosis as seen in Fig. 1. The CT also showed opacification of the middle ear cavity with slight erosive changes of the malleus, as seen in Fig. 2. The chest X-ray showed mild hyperexpansion of the lungs with no evidence of bacterial pneumonia, stable mild to moderate bilateral narrowing of the upper intrathoracic tracheal air shadow, normal heart size, and mild to moderate parahilar peribronchial cuffing indicative of viral infection or bronchitis.

In the ED, the patient was given two 500 mL boluses of intravenous fluids, one dose of 1250 mg of vancomycin, one dose of 2 g of cefepime, 3 mL of ipraptropium bromide/albuterol sulfate every 8 hours, and methylprednisone. The patient was admitted to the inpatient team for antibiotics and surgical intervention. ENT specialist planned for urgent surgical intervention with incision and drainage, complete cortical mastoidectomy and right myringotomy, and tympanostomy tube placement. The patient was admitted for further management of complicated acute right-sided otitis media and comorbidities.

The patient successfully underwent emergent surgery with ENT specialist. The findings included an extensive suppurative process of the mastoid extending from the middle ear to the neck and keratinizing epithelium within the middle ear to mastoid transition (likely cholesteatoma). Cultures were obtained from the mastoid contents, middle ear, and neck abscess.

The patient was initially extubated postprocedure; however, he rapidly developed respiratory distress and acute hypoxic, hypercapnic respiratory failure with resultant reintubation postoperatively. Imaging with both chest X-ray and computed tomography angiography (CTA) chest revealed right bronchiolitis but otherwise unremarkable, and an endotracheal aspirate was sent for culture. Additionally, the patient was noted to have new-onset paroxysmal atrial fibrillation, which initially required amiodarone. However, after conversion to normal sinus rhythm and given the severity of his COPD, the care team elected for rate control with metoprolol tartrate.

The patient’s hospital course was initially complicated by recurrent fevers despite being on vancomycin and cefepime. Therefore, given the concern for inadequate anaerobe coverage, metronidazole was added to the regimen. Additionally, given the concern for extension of the infection to the epidural space and potential inadequate source control, the patient underwent magnetic resonance imaging (MRI) of the cervical spine and repeat CT maxillofacial, which were both nonrevealing beyond postoperative changes.

At 3 days postoperatively, the patient was successfully extubated and had no further respiratory distress noted throughout his hospital stay, with continued oxygen improvement to his home oxygen of 3 L by hospital day five. Throughout treatment, the patient’s blood cultures and endotracheal aspirate remained negative. However, operative cultures from the abscess ultimately grew *Fusobacterium nucleatum*, *Prevotella oris*, and *Parvimonas micra* (formerly *Peptostreptococcus*), and following the infectious disease team’s recommendations, antibiotics were narrowed to ampicillin-sulbactam at 3 g every 6 hours with an intended 2-week intravenous course followed by 2 weeks of oral amoxicillin/clavulanate. Given the organism that grew and the patient’s poor dentition, which revealed tenderness to palpation, a follow-up dental evaluation was recommended. The patient was discharged on postoperative day eight to a skilled nursing facility to complete the antibiotic course, with outpatient follow-up pending.. The patient has been riding his bike, walking short distances, regaining his appetite, and is recovering well.

## Discussion

Since the institution of antibiotics and the incorporation of the pneumococcal conjugate vaccine into routine immunization schedules, the incidence of otitis media has greatly decreased, thereby reducing the incidence of acute coalescent mastoiditis and its accompanying complications. More specifically, the overall fatality rate from intracranial complications has decreased from 35% to 5% [3]. However, the incidence of chronic mastoiditis caused by cholesteatoma has not decreased with antibiotic use [7]. One case study found that in patients with Bezold’s abscesses, 53% presented with erosion of the mastoid tip and 40% presented with a concomitant cholesteatoma [10]. The presence of a cholesteatoma, as present in our patient, can block the outflow of secretions through the external meatus and allow the infectious process to find a weak point at the tip of the mastoid resulting in a Bezold abscess [5]. Additionally, cholesteatomas worsen the ventilation of the ear cavities promoting recurrent superinfections [10]. A Bezold abscess is a rare (only 100 reported cases) complication of acute coalescing mastoiditis in the modern era. These abscesses occur as a result of a progression of the infection through the lateral mastoid complex medial to the attachment of the sternocleidomastoid [1, 3]. If the abscess is not treated properly, the Bezold abscess can descend along the great vessels to reach the perivisceral space, larynx, mediastinum, or retropharyngeal space [5]. In our case, the patient had postoperative respiratory distress and bronchiolitis that likely could have resulted from the descent of the Bezold Abscess.

Anaerobes are a predominant component of the oropharyngeal mucous membrane, thus are frequently associated with head and neck infections, such as chronic otitis media, chronic sinusitis, chronic mastoiditis, head and neck abscesses, cervical adenitis, parotitis, and postoperative infection [7]. In our case, the patient’s poor dental hygiene and the bacteria being anaerobic make dental origin the most likely source of infection. The high prevalence of anaerobes in these complications may suggest a significant role in these bacteria producing a more significant infection that does not respond to ordinary treatment regimens [7]. These anaerobic bacteria can survive penicillin therapy and shield penicillin-susceptible pathogens from the drug, making them difficult to treat and leading to complications. Other case reports document immunocompetent patients developing Bezold’s abscesses as a result of inadequate antimicrobial therapy for acute otitis media [2, 3, 7]. In our case, the patient was prescribed otofloxacin ear drops, which have poor activity against anaerobic bacteria. The inadequate coverage combined with the cholesteatoma likely caused the infection to progress into mastoiditis with a complication of a Bezold abscess. Thus, on initial presentation of otitis media, it is crucial to consider sampling the purulent material for later replacement of initial broad-spectrum antibiotics with ones to which the pathogen has known susceptibility.

## Conclusion

Bezold’s abscess is a rare complication of mastoiditis, with less than 100 cases reported in literature to date. The presence of a cholesteatoma, as seen in our patient, can contribute to the development of both mastoiditis and Bezold’s abscess. Prompt recognition is crucial, as propagation of infection can occur with further tracking along the great vessels and reach the mediastinum, thyroid, or intracranially. Physicians, especially internists, should be aware of this entity, especially given its ramifications on antibiotic selection and the importance of prompt surgical intervention.

## Data Availability

Not applicable.

## Abbreviations

COPD: Chronic obstructive pulmonary disease
ED: Emergency department
BNP: B-type natriuretic peptide
IVF: Intravenous fluids
ENT: Ear, nose, and throat
CT: Computed tomography
CTA: Computed tomography angiography
MRI: Magnetic resonance imaging
IV: Intravenous
PICC: Peripherally inserted central catheter
